# Assessment of knowledge and compliance to evidence-based guidelines for VAP prevention among ICU nurses in Tanzania

**DOI:** 10.1186/s12912-021-00735-8

**Published:** 2021-10-25

**Authors:** Vicent Bankanie, Anne H. Outwater, Li Wan, Li Yinglan

**Affiliations:** 1 XiangYa school of Nursing, Central South University, No.172Tongzi po Road, Changsha, Yuelu District China; 2 Department of Clinical Nursing, University of Dodoma, Dodoma, Tanzania; 3 Department of Community Health Nursing, Muhimbili University of Health and Allied Sciences, Dar es Salaam, Tanzania

**Keywords:** Ventilator-associated pneumonia, Ventilator-associated pneumonia bundle, Evidence-based guidelines, Evidence-based practices, Hospital-acquired infection

## Abstract

**Background:**

Implementation of evidence-based guidelines (EBGs) related to VAP is an effective measure for the prevention of ventilator-associated pneumonia (VAP). While low knowledge regarding the EBGs related to VAP prevention among ICU nurses is still a major concern among nurses in ICUs globally, the situation in Tanzania is scarcely known. This study aimed to assess the ICU nurses’ knowledge, compliance, and barriers toward evidence-based guidelines for the prevention of VAP in Tanzania.

**Methods:**

A cross-sectional study, involving ICU nurses of major hospitals in Tanzania, was conducted. A structured questionnaire was administered among 116 ICU nurses. Data analysis included descriptive statistics and the independent sample t-test.

**Results:**

The mean knowledge score was 3.86(SD = 1.56), based on ten questions (equivalent to 38.6%). Nurses with a degree or higher level of nursing education performed significantly better than the nurses with a diploma or lower level of nursing education (*p* = 0.004). The mean self-reported compliance score for EBGs for the prevention of VAP was 15.20 (SD = 0.93) which is equivalent to 60.8% based on 25 questions. The main barriers to the implementation of EBGs for VAP prevention were lack of skills (96.6%), lack of adequate staff (95.5%), and lack of knowledge (79.3%).

**Conclusion:**

Considering the severity and impact of VAP, and the higher risks of HAIs in resource-limited countries like Tanzania, the lower level of knowledge and compliance implies the need for ongoing educational interventions and evaluation of the implementation of the EBGs for VAP prevention by considering the local context.

## Introduction

In hospitals, Intensive Care Units (ICUs) are among the leading wards in the rate of hospital-acquired infections (HAI) [1, 2]. Patients in hospitals in low-income countries are at higher risk of HAI than patients in hospitals in high-income countries. In a review, the pooled incidence density of ICU-acquired HAI in low-income countries was 47.9 per 1000 patient–days compared to 13.6 per 1000 patient–days in the United States [3].

Ventilator-Associated Pneumonia (VAP) is one of the most common and fatal HAIs in ICUs [3–5]. It is defined as ‘nosocomial pneumonia in ventilated patients that develops more than 48 h after initiation of mechanical ventilation, characterized by a new or progressive infiltrate, fever, altered white blood cell count, and purulent tracheobronchial secretions [6].

Although the exact attributable mortality related to VAP is difficult to ascertain, VAP has long been associated with prolonged ICU stays and increased hospital costs globally [7]. While there is inadequate information regarding VAP incidence in Tanzania and other African countries, the higher burden of infectious diseases and limited resources for treatment and rehabilitation predispose these countries to increased VAP prevalence [5, 8]. Implementation of evidence-based guidelines (EBGs) related to VAP serves as an effective measure for the prevention of ventilator-associated pneumonia (VAP) [9].

To prevent VAP more reliably and effectively, a group of evidence-based interventions called a “VAP bundle” is recommended to help clinicians deliver bedside care [10]. Ventilator-Associated Pneumonia Bundle (VAP bundle) is a series of evidence-based interventions that when implemented together will achieve significant outcomes of reducing VAP in patients on mechanical ventilation [10, 11]. The VAP bundle components include the following [10, 12]: Elevation of the head of the bed (at 30^o^ to 45^o^), daily “Sedation Vacations” and assessment of readiness to be extubated, daily oral care (with chlorhexidine for post-cardiac surgery), peptic ulcer disease (PUD) prophylaxis, and deep venous thrombosis (DVT) prophylaxis [10]. Although there has been some discordance regarding specific bundle components among some researchers [13, 14], VAP reduction has been achieved when the compliance of the main bundle components is achieved [11, 15], provided that there is a high level of compliance (above 95%) to all components of the bundles, unless there is a clear reason for clinical variance, and the reasons are clearly documented [16]. At such high compliance, VAP can be effectively prevented, as revealed in a recent study in the Democratic Republic of Congo, where improving compliance of the VAP bundle components from 0 to 32.75% lowered the VAP incidence density from 33.74 to 18.05 VAP cases per 1000 days on the ventilator [17].

To attain high compliance, nurses have to be well equipped with appropriate knowledge and skills to EBGs as necessary factors for their implementation.

## Background

Although knowledge does not necessarily reflect practice, it remains the first step in the implementation of evidence-based practices. The biggest barrier to compliance with evidence-based practice is not that nurses disagree with the evidence, but rather that nurses do not know whether the evidence exists or do not know what they should be doing [18]. Being the closest patient care providers, nurses in ICU need to have knowledge on the prevention of various hospital-acquired infections for better care of the patient.

Knowledge regarding the EBGs related to VAP prevention is a global concern among nurses in ICUs [4, 19–21], and differs from country to country. The mean knowledge scores reported in various studies range between 41.2% among ICU nurses during the annual Congress of the Flemish Society for Critical Care Nurses [22] and 78.1% among ICU nurses in a tertiary care university hospital in the USA [23]. Although Low- and Middle-Income Countries (LMICs) are more burdened with ICU-acquired HAIs, the knowledge and skills among clinicians in preventing the HAIs is lower than in high-income countries (HICs). In a study to assess the knowledge regarding the EBGs for VAP prevention among nurses, doctors, and respiratory therapists in the USA, all groups had high knowledge about the EBGs for VAP prevention, and intergroup differences in knowledge were not significant [23]. This was contrary to studies done in Egypt [24] and Ethiopia [25] that involved nurses alone, where nurses demonstrated inadequate knowledge.

In addition to the higher rates of infectious diseases in Africa, studies regarding VAP and EBGs for VAP prevention are scarce [3]. In Tanzania, like other sub-Saharan African Countries [3], ICUs are not without risks for VAP. The Majority of patients admitted in adult ICUs present the risk factors reported by Wu et al. [26]. While prolonged hospitalization and other VAP-related complications are frequently reported, there is no clear documentation of VAP incidences and prevalence. Low nurse-patient ratio, high workload, lack of clear and contextualized VAP protocol, limited strictness in maintaining asepsis during aseptic techniques, and limited access to online internet resources are common observable risk factors. The number of specially educated critical care nurses and nurses who have attained specialized ICU knowledge for VAP prevention is also limited. However, the knowledge levels of ICU nurses in Tanzania regarding the EBGs for VAP prevention is unclear, and little attention has been paid to the studies regarding knowledge and compliance to EBPs for VAP prevention among nurses, despite the importance of EBP for VAP prevention in improving the quality and safety of patient’s care in ICUs. Although the emphasis is given to universal precautions for infection prevention and control during ICU care, there is no specific national guideline or protocol for VAP prevention in Tanzania. Therefore, among other things, it was imperative to assess nurses’ knowledge and compliance toward EBGs for the prevention of VAP by nurses working in ICUs, to discover the existing gaps. This gap in knowledge and compliance can be the first step for comprehensive interventions such as clinical teaching to improve knowledge and compliance and to influence local policymaking related to VAP prevention. Therefore, we conducted this study to assess knowledge and compliance towards EBGs for VAP prevention among ICU nurses in Tanzania. To our knowledge, this is the first study regarding knowledge and compliance to EBGs for VAP prevention in Tanzania using a standardized international questionnaire and among the few studies conducted in Africa around this topic.

### Research questions and objectives

The main objective of this study was to explore ICU nurses’ knowledge, compliance, and barriers towards EBGs for the prevention of VAP in Tanzania. The specific research questions were as follows:

1. What do ICU nurses know about EBGs for the prevention of VAP in Tanzania?
2. To what extent do ICU nurses adhere to EBGs for the prevention of VAP in Tanzania?
3. What are the barriers towards EBGs for the prevention of VAP in Tanzania?

## Methods

### Design

A cross-sectional study, with a quantitative approach, was conducted among ICU nurses from all major hospitals in Tanzania.

### Sample size calculation and sampling

The minimum sample size for the study was calculated by using the sample size formula when the mean score is the measure of interest in the study [27] as follows:

N = Z^2^xSD^2^/E^2^.

N = Desired number of participants in a sample.

Z = Standardized value for the corresponding level of confidence. At 95% CI, it is 1.96, E = Margin of error or rate of precision, which was set at 0.25 in this study.

SD = standard deviation, which is based on a previous study or pilot study. In our study, it was derived from the pilot study, which was 1.08.

The calculation yielded 72 participants.

Therefore the minimum sample size was 72.

After adding the 10% non-response, the total sample size was 80 ICU nurses.

However, the number of all nurses who were available during the data collection was 116 and were all involved in the study.

Convenient sampling was employed in the selection of participant nurses. All ICU nurses who were available during the data collection period and willing to participate were included in this study. The inclusion criteria for the participants were as follows: 1) The participants were bedside healthcare providers to ICU patients and in-service nursing students at St.John’s University of Tanzania who are upgrading their diploma into bachelor degree of Nursing during the period of data collection 2) signed informed consent for participation.

The questionnaires were distributed to a total of 116 nursing staff by the well-trained research assistants with a Master of Science in Nursing (Critical Care and Trauma).

### Study settings and participants

Tanzania, one of the East Africa countries has an approximate population of 60 million. The country is administratively divided into 31 regions, and four zones. Each region has a regional hospital, some providing ICU care to a limited number of patients. During the period of our study, there were four main referral hospitals, which are located in zones so as to serve as tertiary level referral centres, receiving patients from regional Hospitals. These hospitals are Kilimanjaro Christian Medical Centre (KCMC) in the Northern zone, Bugando Medical Centre (BMC) in the Western zone, Mbeya Referral Hospital (MRH) in the Southern highlands zone, and Muhimbili National Hospital (MNH) which serves the coastal zone, as well as the national referral hospital receiving patients from all hospitals in Tanzania, including the three named referral hospitals. In Dar es Salaam, in additional to MNH, there are Muhimbili Orthopaedic Institute, Jakaya Kikwete Cardiac Institute (JKCI) and other private hospitals such as such as Aga Khan, Hurbert Kairuki which are also providing long term ICU care.

The participant ICU nurses were recruited from all hospitals in Dar es salaam, which were providing ICU care services during the period of data collection. These hospitals included Muhimbili National Hospital (MNH), Muhimbili Orthopaedic Institute (MOI), Hurbet Kairuki Hospital, and Aga Khan Hospital. In addition, ICU nurses from other regions of Tanzania were conveniently obtained at St. John’s University in Dodoma where they were upgrading their diploma into degrees through bachelor studies. This included ICU nurses from Kilimanjaro Christian Medical College (KCMC), Bugando Medical Centre (BMC), Mbeya Zonal Hospital, and Dodoma Regional Referral Hospital. In overall, the participants came from 10 ICUs, of which 6 ICUs are found in Dar es Salaam, and 4 from other regions (Table 1). The ICUs include orthopedic, cardiac, emergence, and general ICUs. The general ICUs are in some hospitals where all patients requiring ICU care are admitted in a single ICU irrespective of the type of disease condition they are suffering. No participant identification information was required, and the questionnaire was filled only once.

**Table 1:** Demographic characteristics of participant ICU nurses

Variable	Frequency	Percent
**Age (*n*** **= 115)**		
20–29	32	27.8
30–39	59	51.3
40–49	23	20
50–59	1	0.9
**Level of nursing education (*N*** **= 115)**		
Diploma and below	86	74.1
Degree and above	30	25.9
**ICU working experience**		
5 years or less	89	78.1
Above 5 years	25	21.9
**Sex (*N*** **= 116)**		
Male	20	17.2
Female	96	82.8
**Hospital (*N*** **= 116)**		
MNH^a^	53	45.7
MOI^b^,	25	21.6
Kairuki	3	2.6
Aga Khan	14	12.1
St.John’s University^c^,	21	18.1
**Type of ICU (*N*** **= 116)**		
General ICU	61	52.6
Cardiac ICU	10	8.6
Orthopedic ICU	25	21.6
Emergency	20	17.2
**Ever heard about VAP (*N*** **= 116)**		
Yes	59	50.9
No	57	49.1
**Ever cared for a VAP patient(*N*** **= 116)**		
Yes	41	35.3
vNo	75	64.7
**Had any recent course(*N*** **= 114)**		
Yes	18	15.8
No	96	84.2

^a^Muhimbili National Hospital

^b^MuhimbiliOrthopaedic Institute,

^c^Includes KCMC, Bugando Medical Centre, Mbeya Zonal Hospital, and Dodoma Regional Referral Hospital. These nurses were recruited from St. Johns University and participate in ICU care in the St.John’s university-affiliated hospitals

### The questionnaire

The questionnaire was adopted from Jasson et al. (2013) and was previously applied in Finland in 2013 for assessment of knowledge and compliance toward evidence-based practices for prevention of VAP among the critical care nurses [28]. The adopted questionnaire constituted questions from two international pre-validated questionnaires used for assessment of knowledge [29], and compliance and barriers [30]. The knowledge questionnaire comprised of nine closed-ended questions with a difficulty and discriminative indexes of 0.1—0.9; discrimination 0.10—0.65 respectively [31] and supplemented by one question on the use of 0.12% chlorhexidine gluconate antiseptic rinse [13], making a total of 10 objective questions.

The overall Self-reported compliance questionnaire included questions from three main sources:

i) The original questionnaire by Ricart et al. (2003) (question 1–12, 30].
ii) The supplementary questions from the American Association for Respiratory Care (AARC, 2010) recommended open endotracheal suction (ETS) practices (question 13–20) [32], and.
iii) The World Health Organization (WHO, 2009) recommended hand hygiene practices (question 21–25).

Assessment of barriers to implementation of the EBGs for VAP prevention was done according to Jasson et al. (2013). The barriers were outlined and participant nurses were required to either agree by selecting ‘Yes’ or disagree by selecting a ‘No’ option. The percentages of the responses were tabulated and compared.

The overall questionnaire for the assessment of knowledge, compliance, and barriers was evaluated by two experts for reliability: One is a registered nurse with a master of critical care nursing, and another is an anesthesiologist. These experts had ICU working experience of 6 and 9 years respectively.

The overall questionnaires were further pre-tested for internal validity by a group of ICU nurses (*n* = 12) who were not included among participants for the main study. This would reveal whether participant nurses had a common understanding of the questions and whether they could report any ambiguity (actual or perceived) on any of the questions in the questionnaire [33]. This was important so that any differences in participants’ knowledge scores during the actual research could be ascribed to lack of knowledge than failure to understand the question. Therefore, printed questionnaires were distributed to 12 nurses, 4 with the diploma, 2 with the certificate, 4 with the degree, and 2 with the master of nursing, and each nurse was required to answer the questions and give his/her views on the clarity of each question. Generally, the questionnaire was clear and well-understood by the participants.

### Data analysis

Raw data were uploaded and analyzed using Statistical Package for the Social Sciences (SPSS) version 20. Descriptive statistics such as frequency and percentages were used to describe the demographics, compliance, and barriers of participant nurses regarding the implementation of EBGs for VAP prevention. In assessing knowledge, each correct answer was given one mark, thus the total score ranged from 0 to 10. To minimize subjectivity in reporting for compliance, the participant nurses were required to estimate the number of times they adhere to each particular item in every 10 indications for each item. Therefore, in each item, a total of 10 points was given if an item is always and correctly adhered to, and the least score was 0 if the item is not adhered to at all. This allowed an estimate of the compliance rate for each particular item even when the compliance was not 100% per a given item. The total score ranged from 0 to 250. The percentage score for each item was calculated, and the overall compliance score was reported in percentage (Table 3).

Mean and standard deviation was calculated as the measure of central tendency for continuous variable such as knowledge scores. The mean knowledge and compliance scores of ICU nurses were compared by different levels of ICU experiences (≤5 yrs. vs. > 5 yrs) and nursing education (below degree vs degree and above) using an independent sample t-test. Pearson correlation was employed to ascertain the correlation (r) between knowledge and compliance scores to VAP prevention. The interpretation of the correlation coefficient was as follows [34]: 0.00–0.1 negligible correlation,0.1–0.39, weak correlation,0.4–0.69, moderate correlation, and 0.70–0.89 strong correlation and 0.90–1.00, very strong correlation. A *p-value* of less than 0.05 was considered statistically significant.

## Results

### Demographic characteristics of participants

A total of 116 ICU nurses were involved in the study. Nurses aged between 31 and 39 years constituted the largest proportion (42.6%). Most had acquired nursing education at the diploma level and below (74.1%), often with < 5 years of experience (78.1%). About 35% acknowledged having ever cared for a patient with VAP. Only about 16% (*n* = 114) reported having attended an in-service training on VAP. Demographic information of the participants has been summarized in Table 1.

### Knowledge

**Fig. 1:**
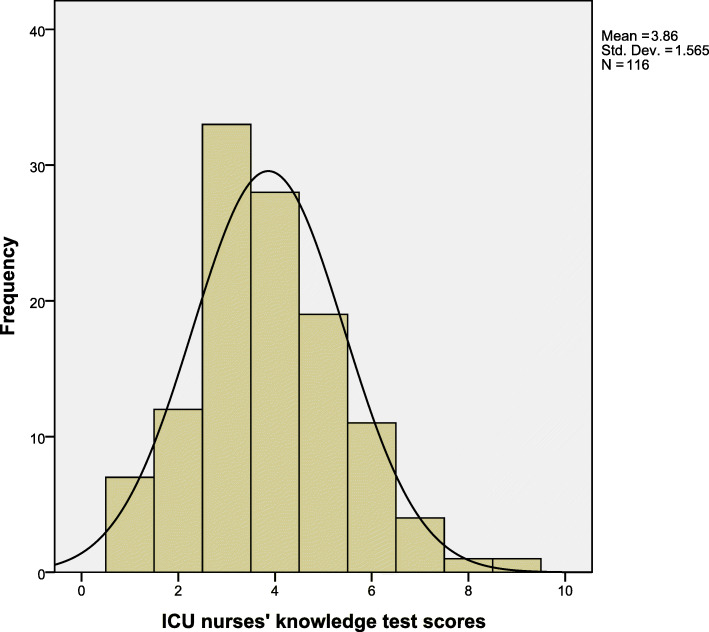
Participant ICU nurses score for the knowledge test

The mean knowledge score (Fig. 1) was 3.86(SD = 1.56), equivalent to 38.6%. Thirty-one per cent achieved more than half of the total points. The scores ranged from 1 to 9 (10–90%). The 75th percentile was a 50% score.

Independent sample t-test revealed that nurses with a higher level of nursing education (degree and above) scored significantly higher than their colleagues with the diploma and below (4.57, SD = 1.22vs 3.62, SD = 1.60, *p* = 0.001) (Table 4). However, although more experienced nurses (ICU experience > 5 years) scored slightly higher than their less experienced colleagues (ICU experience < 5 years), the differences in score was not statistically significant (3.96, SD =1.27, vs 3.86, SD = 1.66, *p* = 0.1).

The top three items to which nurses answered correctly were related to patient positioning (70.7%), oral vs. nasal route for endotracheal intubation (55.2%), use of 0.12% chlorhexidine gluconate antiseptic oral rinse (52.6%)(Table 2). The three least scored items were related to Frequency of humidifier changes (12.9%), type of airway humidifier (23.3%), and Open vs. Closed suction systems (28.4%)(Table 2).

**Table 2:** The responses provided by ICU nurses (*n* = 116) to multiple-choice questions regarding VAP prevention

Questions	Answersn(%)
**Oral vs. nasal route for endotracheal intubation**	
*Oral intubation is recommended*	64 (55.2)
Nasal intubation is recommended	28 (24.1)
Both routes of intubation can be recommended	17 (14.7)
I do not know	7 (6)
**Frequency of ventilator circuit changes**	
It is recommended to change circuits every 48 h (or when clinically indicated)	45 (38)
It is recommended to change circuits every week (or when clinically indicated)	29 (25)
*It is recommended to change circuits for every new patient (or when clinically indicated)*	38 (32.8)
I do not know	4 (3.4)
**Type of airway humidifier**	
Heated humidifiers are recommended	38 (32.8)
*Heat and moisture exchangers are recommended*	27 (23.3)
Both types of humidifiers can be recommended	41 (35.3)
I do not know	10 (8.6)
**Frequency of humidifier changes**	
It is recommended to change humidifiers every 48 h (or when clinically indicated)	74 (63.8)
It is recommended to change humidifiers every 72 h (or when clinically indicated)	23 (19.8)
*It is recommended to change humidifiers every week (or when clinically indicated)*	15 (12.9)
I do not know	4 (3.4)
**Open vs. closed suction systems**	
Open suction systems are recommended	33 (28.4)
Closed suction systems are recommended	29 (25)
*Both systems can be recommended*	33 (28.4)
I do not know	21 (18.1)
**Frequency of change in suction systems**	
Daily changes are recommended (or when clinically indicated)	40 (34.5)
Weekly changes are recommended (or when clinically indicated)	26 (22.4)
*It is recommended to change systems for every new patient (or when clinically indicated)*	36 (31.0)
I do not know	14 (12.1)
**End otracheal tubes with extra lumen for drainage of subglottic secretions**	
*These endotracheal tubes reduce the risk for VAP*	56 (48.3)
These endotracheal tubes increase the risk for VAP	30 (25.9)
These endotracheal tubes do not influence the risk for VAP	21 (18.1)
I do not know	9 (7.8)
**Kinetic vs. standard beds**	
Kinetic beds increase the risk for VAP	30 (25.9)
*Kinetic beds reduce the risk for VAP*	39 (33.6)
The use of kinetic beds does not influence the risk for VAP	35 (30.2)
I do not know	12 (10.3)
**Patient positioning**	
Supine positioning is recommended	17 (14.7)
*Semi-recumbent positioning is recommended*	82 (70.7)
The position of the patient does not influence the risk for VAP	12 (10.3)
I do not know	5 (4.3)
**Use of 0.12% chlorhexidine gluconate antiseptic oral rinse**	
*0.12% chlorhexidine gluconate antiseptic oral rinse reduce the risk of VAP*	61 (52.6)
0.12% chlorhexidine gluconate antiseptic oral rinse increase the risk of VAP	33 (28.4)
0.12% chlorhexidine gluconate antiseptic oral rinse does not influence the risk of VAP	20 (17.2)
I do not know	2 (1.7)

Mean score = 3.86, 38.6%

SD = 1.57,15.7

Having a recent course on VAP, or ever cared a VAP patient did not significantly affect the knowledge on EBGs for VAP prevention (Table 4).

### Compliance

The mean self-reported compliance score for EBGs for prevention of VAP (Table 3) was 15.20 (SD = 0.93) which is equivalent to 60.8%.

**Table 3:** Intensive Care Unit nurses’ self-reported compliance to EBGs for prevention of VAP

	%(*n* = 116)
Removal of the nasogastric tube as soon as clinically feasible	81.6
Enteral feeding protocol/avoidance of gastric over distension	91.2
Semi-recumbent positioning of the patient (30—45°)	89.2
Humidification with heat and moisture exchangers	84.1
Daily changes of heat and moisture exchangers	59.1
Chest physiotherapy	55.5
Adequate hand hygiene between patients	87.5
Use of a formal infection-control program	90.6
Maintenance of adequate pressure in the endotracheal-tube cuff	82.1
Scheduled drainage of condensate from ventilator circuits	29.6
Continuous subglottic suctioning	11.6
Use of protective gowns during suctioning	11.6
Pre-suctioning analgesic	0.4
Pre-suctioning hyperoxygenation	41.1
Face mask-wearing during suctioning	11.6
Sterility of suction catheter maintained until inserted into airway	90.8
Protection of patients eyes and central venous catheter from secretions during suctioning	10.9
Two nurses perform suctioning	13.0
Sodium chloride instillation	90.5
Used catheter and gloves are disposed of in a manner that prevents contamination from secretions	90.1
Sedation protocol	75.3
Respirator and weaning protocols	60.1
Avoidance of unnecessary reintubation	89.7
Extubation protocol	84.0
Patient positional treatment	91.8

Mean compliance was 60.8 (SD = 3.8),

Independent sample t-test revealed that experience and educational levels had no significant association with self-reported compliance to EBGs for VAP prevention (Table 4).

**Table 4:** Relationship between knowledge, compliance and selected demographic characteristics

Test variable	Grouping	Mean (SD)	t-value	***P***-value
**Knowledge**	**Experience**			
	Experience> 5	3.96 (1.27)	0.343	0.1
	Experience< 5	3.85 (1.66)		
	**Education**			
	Degree and above	4.57 (1.22)	2.96	**0.004**
	Diploma and below	3.62 (1.60)		
	**Ever cared a VAP patient**			
	Yes	4.02 (1.28)	0.897	0.37
	No	3.77 (1.71)		
	**Had any training regarding VAP**			
	Yes	3.61	0.904	0.37
	No	3.91		
**Compliance**	**Experience**			
	Experience> 5	15.02 (0.6)	1.706	0.09
	Experience< 5	15.30 (1.0)		
	**Education**			
	Degree and above	15.27	0.758	0.74
	Diploma and below	15.14		
	**Ever cared a VAP patient**			
	Yes	15.21 (1.30)	0.206	0.81
	No	15.25 (0.66)		
	**Had any training regarding VAP**			
	Yes	15.12 (0.61)	0.839	0.407
	No	15.26 (0.99)		

The three most adhered procedures were related to semi-recumbent positioning of the patient (92.4%), patient positional treatment (91.8%), and enteral feeding protocol/avoidance of gastric overdistension (91.2). The four least adhered procedures were related to Pre-suctioning analgesic (0.4%), use of protective gowns during suctioning (11.6%), face mask-wearing during suctioning (11.6%), continuous subglottic suctioning (11.6%).

Having a recent course on VAP, or ever cared a VAP patient with VAP did not significantly affect the compliance to EBGs for VAP prevention.

### Correlation between knowledge and compliance

The correlation coefficient between knowledge and compliance was 0.01, (*p* > 0.05).

### Barriers

The main reported barriers to implementation of EBGs for VAP prevention (Table 4) include Lack of skills (96.6%), Lack of staff (95.7%), and Job discretion (94%). The least reported barriers include the procedures considered unnecessary (4.3%), (Table 5).

**Table 5:** Barriers to EBG related to VAP

Barrier	n (%)
Lack of skills	112 (96.6)
Inadequate staff	111 (95.7)
Job discretion	109 (94)
Insufficient knowledge	92 (79.3)
Lack of guidance	91 (78.4)
Laziness	87 (75)
Considered unnecessary	5 (4.3)

## Discussion

This study aimed at determining knowledge, compliance, and barriers to implementation of EBGs for VAP bundle prevention in Tanzania as a resource-limited setting. The mean knowledge score was 3.86(SD = 1.56), which is equivalent to 38.6%. This score is below the mean scores ever reported in various studies, ranging from 41.2% among nurses during the annual congress of the Flemish Society for Critical Care Nurses in November 2005 [22] to 78.1% in the USA [23]. Poor knowledge regarding the EBGs related to VAP prevention has also been reported in Iran [19], Yemen [21] and Taiwan [35] in Asia, and Egypt [24] and Ethiopia [25] in Africa. The differences in knowledge scores may be explained by the differences in models of healthcare delivery in ICUs [23], and lack or differences in specific guidelines and policies regarding training and practice of EBGs for VAP prevention in ICUs [29]. Developing a specific guideline and policy for training VAP prevention by considering the challenges in the resource-limited setting, without compromising the effectiveness in VAP prevention, could be helpful in minimizing the knowledge differences in resource-limited settings. Such standardized guidelines would take into consideration the costs related to recommendations for VAP prevention.

This study reveals a higher range of knowledge among nurses (10–90%) not only among ICU nurses in different hospitals but also within the same hospital. This higher range in knowledge between the lowest and the highest knowledge score may imply the difficulty in sharing evidence-based information among staff. The difficulty in sharing knowledge in hospitals in resource-limited settings has been extensively documented [36, 37]. Some associated factors to information sharing include Differences in educational levels, limited resources, job dissatisfaction, lack of motivation, and lower level of professional education [36, 37]. Other factors may include high workload, lack of organized on-the-job training, and lack of emphasis to improve knowledge or practice regarding EBGs. Yonkaitis and Maughan [38] have provided a simplified and useful guide for EBG knowledge sharing and evaluation (the 6 ‘A’s’ of EBPs) which may be adopted in resource-limited settings to assess the need, acquire the best evidence, appraise the evidence, apply evidence and disseminate evidence [38].

In our study, nurses with a degree or higher level of nursing education performed significantly better than the nurses with a diploma or lower level of nursing education. These results are consistent with studies in Taiwan [35], Ethiopia [25], and Belgium [29] but are contrasted by the study in New Zealand [39]. However, contrary to other studies [22, 23, 29, 30], and consistent with others [28], nursing assistants were included because they are also involved in bedside care of critically ill patients in ICU. However, their proportion was very low (1.7% of the entire sample), and therefore the results should be interpreted with caution.

Our study, unlike several others [22, 29, 40] revealed that there was no difference in knowledge between more experienced nurses and less experienced nurses. These results are consistent with studies in New Zealand [39], Ethiopia [25], and the USA [23]. In resource-limited countries like Tanzania, continuing education programs for in-service nurses are not common. The reliable source of knowledge remains college nursing training. Therefore, nurses with lower nursing education are likely to remain with little knowledge despite their increased clinical experiences, most of which are based on routine works and fulfilling medical orders.

In our study, the mean self-reported compliance to EBGs for the prevention of VAP was 15.20 (SD = 0.93) which is equivalent to 60.8%. This score is below the compliance scores ever reported in various studies, ranging between 77.7% in Spain [30] and 83% in the USA [41]. Consistent with other studies [28], neither nursing level of education nor experience was associated with significant variability in compliance. In a similar study in Ethiopia [25], only higher nursing experience was associated with increased compliance to EBGs for VAP prevention. It implies that there are other factors than the nursing level of education, and experience that affect compliance to the EBGs for VAP prevention, which may range from institutional factors such as lack of sufficient management support and policy to individual factors such as heavy workload and increased job stress in a resource-limited setting.

Consistent with other studies [28], the most commonly self-reported compliances were related to semi-recumbent positioning. Others include patient positional treatment, enteral feeding protocol/avoidance of gastric overdistension, use of a formal infection-control program, sterility of suction catheter maintained until inserted into the airway, sodium chloride instillation, and disposal of used catheter and gloves in a manner that prevents contamination from secretions. The reason for the high compliance score could be because these are part of the local guideline for ICU care of critically ill patients in most ICUs in Tanzania, and therefore, are routinely performed. The least adhered component was related to presuctioning analgesia (0.4%), which is much lower than the previously reported studies [28]. In Tanzania, administering presuctioning analgesia is almost not done in ICUs.

In our study, while 28% of the participant responded that both the open and closed systems are recommended, 25% responded only closed systems are recommended. In general, both the open and closed suction systems have similar results in terms of safety and effectiveness in preventing HAI [42]. However, a closed-suction system is desirable and is likely to add extra protection against COVID-19 transmission in ICU [43]. Sunctioning is an aerosol-generating procedure on critically ill patients with COVID-19 and present an increased risk of contamination for ICU nurses. With a massive number of critically ill patients admitted due to COVID-19, and irrespective of the full personal protective equipment that nurses wear, the open tracheal suction technique (OTST) still represents a potential threat for nurses [43]. Therefore, Closed tracheal suction systems (CTSS) remains a safe sunction method which allows the removal of tracheobronchial secretions without disconnecting ventilatory circuits, gas exchange deterioration and hypoxia to patients, and should be emphasized to ICU nurses in their daily ICU care.

The main barriers to the implementation of EBGs for VAP prevention were lack of skills (96.6%), lack of staff (95.5%), and lack of knowledge (79.3%). These factors are also reported in several other studies [41, 44]. Lack of knowledge and skills may be attributed to the inability to transform research into practice, and poor information sharing among nurses as the majority of ICU nurses have lower nursing education levels [23, 44]. Poor information sharing among ICU nurses is revealed by a wider range of knowledge scores of 80% (10–90%) in the present study. Others include lack of guidance (78.4%), and laziness (75%) [28].

Our study reveals that there is no correlation between knowledge and compliance(r = 0.01, *p* > 0.05). This is consistent with other studies [24, 45] which also revealed lack of association between knowledge and practice regarding VAP prevention. In contrast, other educational interventional studies reveals that, an increased knowledge, improves compliance which also reduces VAP incidences [46]. The differences among the studies may be due to the differences in the type and extent of barrier factors that may impede compliance despite increased knowledge. Although knowledge remains the first step in the implementation of EBGs for VAP prevention, other factors such as the limited number of staff and lack of managerial support may affect compliance despite increased knowledge [28].

In summary, it is necessary that the knowledge, compliance, and barriers are assessed so that measures are taken for the improvement of clinical outcomes of our ICU patients. The knowledge levels and compliance of ICU nurses in Tanzania regarding EBGs for VAP prevention are lower than the lowest ever reported level of knowledge in the published studies. This may be the single most important barrier to the implementation of the EBGs for VAP prevention.

### Implication and recommendation for practice

Considering the implication of VAP in the quality of ICU patients, and the role of compliance to the EBGs in the prevention of VAP and improving quality of ICU patient care, educational measures to improve knowledge, preparing standardized guidelines, and enhancing information sharing among nurses may have significant outcomes in the prevention of VAP. Using strategies such as shift–based educational interventions and bedside teaching, nurses with higher knowledge and experience may be important agents in improving knowledge and compliance to other nurses regarding VAP prevention.

Whenever possible, increasing the nurse to patient ratio in ICUs will add to the implementation of the recommended EBGs for VAP prevention. The results of this study will help in guiding local practice and education and will be the baseline of reference after the implementation of educational measures. Furthermore, being the first study regarding knowledge and compliance to EBGs for VAP prevention in Tanzania using a standardized international questionnaire, the results of this study add to the existing literature regarding the state of sub-Saharan Africa and other resource-limited settings.

### Limitations

This study had some limitations worth mentioning. First, the participant ICU nurses outside Dar es Salaam City were conveniently obtained at St. John’s University. These may not be representative of all other nurses in their respective hospitals. Second, this study did not exhaustively evaluate other factors that may affect compliance, such as managerial factors.

Third, our study evaluated self-reported compliance which may differ from the observed (actual) compliance to EBGs for VAP prevention among the ICU nurses.

## Conclusion

The average knowledge level and compliance regarding the EBGs for VAP prevention in Tanzania was lower than the lowest ever reported elsewhere. The level of nursing education was shown to be associated with better knowledge scores. Barriers towards EBGs were identified. There is a need for ongoing in-service educational interventions and effective implementation strategies. Strong mentorship and exchange programs across the country on knowledge and skills transfer among ICU nurses in Tanzania are highly recommended. Considering the consequences of VAP, Nursing curriculums at all levels should include a part for EBGs for VAP prevention.

## Data Availability

Data for the study are available to the corresponding author and may be accessed upon request.
